# The creation and evaluation of a model to simulate the probability of conception in seasonal-calving pasture-based dairy heifers

**DOI:** 10.1186/s13620-017-0110-0

**Published:** 2017-11-22

**Authors:** Caroline Fenlon, Luke O’Grady, Stephen Butler, Michael L. Doherty, John Dunnion

**Affiliations:** 1 UCD School of Computer Science, University College Dublin, Belfield, Dublin 4, Ireland; 2 UCD School of Veterinary Medicine, University College Dublin, Belfield, Dublin 4, Ireland; 3 Animal and Grassland Research and Innovation Centre, Teagasc, Moorepark, Fermoy, County Cork, Ireland

**Keywords:** Dairy cow, Fertility, Conception, Nulliparous heifer, Machine learning, Regression, Simulation, Discrimination, Calibration

## Abstract

**Background:**

Herd fertility in pasture-based dairy farms is a key driver of farm economics. Models for predicting nulliparous reproductive outcomes are rare, but age, genetics, weight, and BCS have been identified as factors influencing heifer conception. The aim of this study was to create a simulation model of heifer conception to service with thorough evaluation.

**Methods:**

Artificial Insemination service records from two research herds and ten commercial herds were provided to build and evaluate the models. All were managed as spring-calving pasture-based systems. The factors studied were related to age, genetics, and time of service. The data were split into training and testing sets and bootstrapping was used to train the models. Logistic regression (with and without random effects) and generalised additive modelling were selected as the model-building techniques. Two types of evaluation were used to test the predictive ability of the models: discrimination and calibration. Discrimination, which includes sensitivity, specificity, accuracy and ROC analysis, measures a model’s ability to distinguish between positive and negative outcomes. Calibration measures the accuracy of the predicted probabilities with the Hosmer-Lemeshow goodness-of-fit, calibration plot and calibration error.

**Results:**

After data cleaning and the removal of services with missing values, 1396 services remained to train the models and 597 were left for testing. Age, breed, genetic predicted transmitting ability for calving interval, month and year were significant in the multivariate models. The regression models also included an interaction between age and month. Year within herd was a random effect in the mixed regression model. Overall prediction accuracy was between 77.1% and 78.9%. All three models had very high sensitivity, but low specificity. The two regression models were very well-calibrated. The mean absolute calibration errors were all below 4%.

**Conclusion:**

Because the models were not adept at identifying unsuccessful services, they are not suggested for use in predicting the outcome of individual heifer services. Instead, they are useful for the comparison of services with different covariate values or as sub-models in whole-farm simulations. The mixed regression model was identified as the best model for prediction, as the random effects can be ignored and the other variables can be easily obtained or simulated.

## Background

Herd fertility in pasture-based dairy farms is a key driver of farm economics [1]. The primary goal of reproduction management of both nulliparous heifers and lactating cows is to have animals conceiving in a timely manner to achieve the optimal calving pattern. The factors affecting the reproductive performance of lactating dairy cows have been comprehensively studied, but analyses of heifer fertility have been rare. Careful management of heifer reproduction is important to optimise future production, health and the costs of calf-rearing [2]. It is generally accepted that calving for the first time should occur at 22 to 24 months of age in both seasonal-calving grazing systems [3–5] and year-round calving confinement systems [2, 6] to maximise lifetime productivity.

The reproductive performance of nulliparous animals comprises three major components: the onset of ovarian activity during puberty; expression and detection of oestrus for service; and the outcome of service once performed. The individual effects of age, weight, genetics and management have been identified as significantly impacting the likelihood of conception in the heifer’s first breeding season.

Weight and size (and thus nutrition) throughout the development of young animals are important for their later reproductive performance. Greater average growth rates in weight and girth from 30 to 450 days resulted in younger breeding of UK Holstein-Friesian heifers [7]. Increased weaning weight and post-weaning weight gain increased the probability of Angus and Angus-Hereford beef heifers reaching puberty before the start of breeding [8]. The probability of conception at first service also increased with weaning weight.

Higher body weight and BCS at mating start date (MSD) had a positive effect on the proportions of heifers identified as pubertal in herds of Holstein-Friesian heifers and resulted in earlier calving dates [3]. Donovan et al. [9] found that increased pelvic size and withers height had a positive effect on first service conception in Holstein heifers in Florida. Heifers that were heavier at the start of breeding had increased incidence of oestrus and higher pregnancy rates at the end of the season than lighter animals [10].

Age and weight at first breeding are closely correlated [11]. Greater age at MSD has been found to be positively associated with the proportion of Holstein-Friesian heifers identified as pubertal [3]. Conception rates in groups of maiden heifers calving older than 750 days were lower than in younger heifers, with service failure or delays in cyclicity or breeding identified as causes [2]. Age is particularly important in farming systems with restricted calving periods, with heifers ideally conceiving early in the breeding season [4].

Breed and strain interact with age and weight; North American strains of Holstein-Friesian heifers are older and heavier at puberty than New Zealand Friesian animals [5]. Dairy heifers from a line selected for genetic merit for milk yield and fat/protein yield had poorer conception to first service, a longer interval from first to last service and a greater number of services per conception, compared to a line maintained with UK average yield and fat/protein merit [12].

Few multivariate models have been created to allow prediction of heifer conception. Donovan et al. [9] created a logistic regression model to predict conception at first service. The significant effects were breeding season, oestrus detection method, use of prostaglandin and an interaction between pelvic size and season. Holm et al. used regression to model the effects of age, weight and reproductive tract scoring on pregnancy rate within 50 days. Calendar year, month and heifer age were the most important factors affecting conception rate [13]. Service number and service sire breed, age and inbreeding were also significant, but contributed less to the model. Bergmann and Hohenboken [6] identified the combination of birth date and relative growth rate from weaning to yearling as the best logistic regression model of Angus and Simmental heifer conception within a 63-day breeding season.

Accurate models predicting epidemiological outcomes are useful tools for decision-support and simulation. Validating the predictive ability of the model is an important step in verifying its usefulness. The models described above were evaluated using model deviance [6], goodness-of-fit tests [9] and area under the ROC curve [14]. All of the models were tested on the same data used to train the model. To identify coefficient over- or under-estimation and bias, an independent dataset should ideally be used for model verification [15]. If an external collection of data is not available, resampling techniques such as bootstrapping may be used to aid unbiased model-building. Two types of evaluation are possible for assessing the predictive ability of binary models. *Discrimination* measures the model’s ability to correctly distinguish between positive and negative outcomes and includes tests such as sensitivity and specificity. *Calibration* tests the accuracy of the predicted probabilities with graphical assessment and overall goodness-of-fit tests [16].

The objective of this study was to create and evaluate a predictive model of conception to AI service in seasonal-calving dairy heifers. The primary aim of this model is as a component in the whole-farm simulation of dairy heifers.

## Methods

### Data

Records of AI breeding events performed on both research and commercial Irish dairy farms were used in this analysis. 1216 breeding events from 858 heifers were sourced from the centralised database at Teagasc’s Animal and Grassland Research and Innovation Centre, Moorepark, County Cork, Ireland. The animals included in the dataset were from the Curtins and Ballydague spring-calving research herds from 2001 to 2009, which were representative of Irish grass-based farming systems with a diverse range of cow genetics [17]. An additional 2400 breeding events from 1831 heifers were recorded on ten commercial dairy farms involved in a herd fertility consultancy program operated by the University College Dublin School of Veterinary Medicine [18] from 2009 to 2013. All farms were managed using spring-calving pasture-based production systems. On each farm, animals were served to standing oestrus by a single AI operator. No bull services were included in the records from the research herds. Bull services performed after the initial period of artificial insemination in the commercial herds were managed in the same way as the AI services but were not included in the study.

### Data cleaning and calculations

#### Service dates

All of the service events used in this study were performed from April to July. The details provided for each service were calendar date, service number, days since the previous service and service sire. Service number was categorised with values ≥3 grouped together. The number of days since the previous service (inter-service interval) was grouped into the following bins: ≤ 17; 18–24; 25–35; 36–48; and ≥49 days.

To confirm the outcome of each service, the time between the date of the service and the following calving date was calculated. Where the calving was not identified as an abortion and the proposed gestation length was too short (< 267 days), the gestation lengths of earlier services were tested. If one of the earlier services was more closely aligned with a normal gestation length, it was marked as positive and later services were removed (*N* = 115). If the proposed gestation length was too long (> 300 days), the final service was removed (*N* = 166); it was assumed that the service was either unsuccessful or resulted in embryonic loss followed by another, unrecorded, conceiving service. Final services marked as negative but followed by calving within 282 ± 15 days were changed to positive.

Services occurring less than three days after the previous service, which were assumed to occur during a single oestrus, were removed (*N* = 101). Breeding seasons where animals were repeatedly bred after short intervals were identified as animals with potential ovarian issues and removed from the dataset (*N* = 13). Service numbers and inter-service intervals were recalculated after changes were made. Carryover services for non-pregnant heifers outside of the range of the spring breeding season were removed (*N* = 574).

This analysis concerns the prediction of service success, and thus assumes that the corrected service events were performed at genuine oestrus events.

#### Breed and genetics

Records on percentage primary breeds were available, with Holstein and Friesian being the most predominant. Other breeds were: Friesian cross; Jersey and Jersey cross; Montbéliarde; Normande; Norwegian Red; and other minor breeds. Predicted transmitting ability (PTA) values used in the calculation of the Irish national breeding programme Economic Breeding Index values were available for the heifers in the data set. These included the fertility sub-index and PTAs for calving interval (CIV), survival, milk production, and fat and protein yield and composition. A description of how the individual PTA traits contribute to each of the sub-indexes is available from the Irish Cattle Breeding Federation website [19]. Details on the individual calculation of PTA and economic weightings are described by Berry et al. [20].

### Model-building

All analyses were carried out using the R statistical programming language [21].

An initial univariate analysis was conducted, using logistic regression, to screen candidate variables for inclusion in the multivariate models and to test for non-normal relationships with the dependent likelihoods.

A randomly-selected 70% of the available data was used to train the multivariate models, with the other 30% held back to evaluate the predictive ability of the models. The “createDataPartition” function (from the R caret package [22]) was used to retain equal conception rates across the two datasets. The models without random effects were built using the bootstrapping resampling technique from the same package, with 2000 iterations.

Two different forms of regression analysis were used to model the likelihood of conception to service in heifers. Logistic regression, with and without random effects, is one of the most popular techniques for modelling binary outcomes. Generalised additive models are an extension of regression with fewer assumptions about the data, but reduced interpretability. Both techniques predict the probability of the event occurring, which can then be transformed to a binary outcome using a threshold probability.

#### Logistic regression

Binary logistic regression [16] (R function “glm” [21]) is a generalisation of simple linear regression designed to model the effect of independent variables on the probability of the modelled outcome occurring. Logistic regression assumes all independent variables are normally distributed and not strongly correlated. Regression analysis allows for interactions between independent variables to be included in the model.

The available factors were all considered for inclusion in the multivariate logistic regression model (LR). The model was created using the variables with *P* ≤ 0.2 in the univariate analysis. It was then refined by removing variables not significant with *P* > 0.2 (using the R “drop1” function) and adding variables or interactions of clear biological importance with *P* ≤ 0.05 (“add1” function). This was repeated until no more factors were available to improve the model. Two-way interactions of model components were considered in the same way. To test the linearity of the continuous effects with respect to the logit probability of conception, a model containing only the continuous effects and interactions with their log was created. Significant terms (*P* < 0.05) indicate non-linear relationships.

#### Mixed logistic regression

Random effects can be incorporated into logistic regression to account for the influence of unmeasurable events or global effects. All factors potentially impacting conception in an unquantified way were considered for inclusion as random effects in a mixed logistic regression model (MLR), using the “lme4” R package (Bates, Maechler, Bolker, & Walker, 2015): cow, service sire, year of service and herd.

#### Generalised additive model

Similarly to logistic regression modelling, generalised additive modelling (GAM) can be carried out on dependent variables from a range of distributions. The independent variables may be estimated using smoothing functions, however, thus relaxing the basic regression assumptions about the relationship between the predictors and the dependent variable [23]. Basic generalised additive models do not explicitly model interactions.

### Variable importance

Rankings of the variables included in the bootstrapped logistic regression and generalised additive model were calculated based on the magnitude of each variable’s contribution to the prediction (“varImp” caret package function). The variables in the mixed logistic regression were ranked by the *P*-value for their inclusion in the model.

### Model evaluation

As the aim of this study was to create the best simulation model of heifer conception to service, the predictive ability of the models was evaluated based on discrimination and calibration.

#### Discrimination

Discrimination measures the ability of a model to accurately distinguish between positive and negative outcomes. The probabilities predicted by each model were transformed into binary predictions of conception with the standard discrimination threshold (50%) as the cut-off between negative and positive outcomes. The actual and predicted outcomes were tabulated in a confusion matrix. From this, the discrimination metrics sensitivity, specificity, positive predictive value (PPV) and negative predictive value (NPV) were calculated. The overall prediction accuracy was also calculated. The Matthews correlation coefficient (MCC) was calculated to compare the performance of the models with a random classifier [24]. It ranges from −1 (completely inaccurate predictions) to +1 (completely accurate predictions), with 0 indicating the same performance as a random predictor.

For each model, the receiver operating characteristic (ROC) curve was created by plotting the tradeoff between sensitivity and 1 – specificity as the discrimination threshold is altered (ROCR R package [25]). The area under the ROC curve (AUC) indicates the probability that the model will predict a higher probability for a randomly-chosen positive instance than for a randomly-chosen negative instance. The optimal discrimination threshold was determined from the ROC curves and then used to reclassify the binary predictions and recalculate the other discrimination statistics. Using the optimal threshold minimises classification error when using a model to predict the outcome of a service.

#### Calibration

Calibration measures the accuracy of the model’s predicted probabilities, without consideration of the ultimately predicted outcome [16]. This is typically carried out by grouping similar records and comparing the mean predicted probabilities to the mean rates of occurrence within each group.

The Hosmer-Lemeshow test [26] was used to evaluate the overall goodness-of-fit of the model predictions. The test, performed using the “hoslem.test” function of the ResourceSelection package [27], splits the observations (sorted by predicted probability) into ten equal-sized groups of risk and compares the observed number of events to the mean predicted number of events within each group. The result has a chi-square distribution. The alternative hypothesis of the test is that the model does not fit the data in question correctly.

For each set of model predictions, the observations were grouped into 15 equi-interval bins and the mean predicted probability was plotted against the proportion of true events within each group. The number of bins was chosen to allow for groups of reasonable size, while still maintaining low within-group probability variation. Groups containing less than 10 records were not plotted. Confidence intervals for the proportions in calf were calculated using the F distribution, in the “calibration.plot” function of the PresenceAbsence R package [28]. The “val.prob” function from the rms [29] R package was used to perform a chi-square test to evaluate the “unreliability” of the calibration line: whether the line fitted to the calibration points differed from the 45° line indicating perfect prediction (i.e. intercept = 0 and slope = 1).

The difference between the predicted probability of conception and the true outcome (1 or 0) was calculated for each of the service records, which were then discretised into equal-sized groups sorted by predicted probability. The average group deviances were generated using the “binned.resids” function of the arm package [30]. The absolute group deviance values were averaged to find the mean absolute calibration error (MACE), a measure of overall predictive error.

## Results

After cleaning and the removal of breeding records with missing values, 1993 service records from 1608 heifers remained for use in training and testing the multivariate models. Missing values in breed and calving interval PTA were the primary causes of data loss. Of these services, 1549 (77.7%) resulted in conception. Additional descriptive statistics, displayed by herd, are summarized in Table 1. 1396 randomly selected records were used to build the models, with the remaining 597 records held back for evaluation.

**Table 1:** Descriptive statistics by herd (mean, with SD in parentheses where appropriate)

	Research Herds		Commercial Herds									
	1	2	1	2	3	4	5	6	7	8	9	10
Years	10	10	5	5	5	4	5	5	4	5	4	4
Herd size	42.80 (20.44)	26.60 (21.87)	40.20 (6.06)	11.00 (7.35)	29.80 (9.58)	15.50 (2.89)	14.40 (4.93)	12.00 (5.52)	17.50 (4.20)	28.80 (3.56)	16.25 (6.85)	9.00 (6.00)
Services per lactation	1.27 (0.52)	1.33 (0.65)	1.24 (0.47)	1.18 (0.47)	1.20 (0.43)	1.29 (0.55)	1.31 (0.52)	1.20 (0.40)	1.06 (0.23)	1.10 (0.30)	1.14 (0.39)	1.42 (0.77)
Service conception rate (%)	76.94 (42.16)	73.24 (44.33)	90.76 (29.01)	61.54 (49.03)	72.63 (44.71)	68.75 (46.64)	60.64 (49.12)	88.89 (31.65)	85.14 (35.82)	87.34 (33.36)	78.38 (41.45)	80.39 (40.10)
In calf within 84 d (%)	95.53 (5.59)	97.16 (4.05)	94.32 (3.87)	59.43 (42.01)	78.46 (9.23)	85.30 (11.31)	77.34 (21.08)	89.33 (15.35)	86.57 (10.30)	92.40 (2.71)	88.69 (13.93)	94.64 (6.84)
Age at service (d)	447 (30)	455 (45)	440 (33)	537 (79)	464 (58)	488 (57)	475 (61)	445 (18)	493 (68)	461 (61)	518 (53)	463 (72)
Monthly services (%)												
April	65.65	50.30	37.18	50.00	32.14	26.58	63.22	32.35	5.63	48.05	63.89	40.00
May	27.70	36.14	61.54	7.81	56.55	41.77	22.99	66.18	80.28	50.65	27.78	31.11
June	5.88	12.05	1.28	32.81	10.12	18.99	13.79	1.47	14.08	1.30	8.33	24.44
July	0.76	1.51	0.00	9.38	1.19	12.66	0.00	0.00	0.00	0.00	0.00	4.44

### Univariate results

Age (measured in days) was found to be statistically significant, with an increase of 10 days resulting in a 3% reduction in the likelihood of a successful service. Services carried out in May were 1.6 times more likely to be successful than those in April, but no other months were significantly different. Neither the service number nor inter-service interval showed any statistical significance. Longer CIV PTAs (i.e., poorer merit) resulted in a lower probability of conception, decreasing by 7% for every interval increase of one day. Greater fertility sub-index and survival PTA values also increased conception likelihood. Breed was significant, with heifers of other minor breeds less than half as likely (0.47) to conceive as Holstein heifers at a given service. No other breed categories were significantly different from Holstein in the univariate analysis.

### Multivariate models

The factors identified as significant in stepwise logistic regression analysis of the full dataset were: age; breed; CIV PTA; month; and year. A significant interaction between age and the month of service was included in LR and MLR. The continuous variables in the LR model were confirmed to be linear in respect of the logit likelihood of conception.

Year was instead used as a random effect in the mixed logistic regression model, nested within herd. The mean of every random effect is 0. The standard deviation of the random effect was 0.78.

Variable importance rankings for the three models are presented in Table 2. The GAM modelled a subset of the variables and did not explicitly include interactions. Year was highly important in LR and GAM, with several years in the top five ranking for both. Calendar month was important in all three models. May was the most important value in GAM, while June/July ranked first for MLR and fourth for LR. Breeding events in May were less important for LR and MLR, ranking tenth and fifth, respectively. In GAM, June/July ranked seventh. Age was the eighth most important variable in GAM and ranked 20th and 14th in LR and MLR, respectively. The interaction between age and June/July breeding was third in MLR and fifth in LR, while the interaction with May ranked seventh and 15th in MLR and LR, respectively. Holstein, at position four, was the only breed modelled by GAM. Norwegian Red was the breed with the most importance in LR (sixth) and MLR (fourth). Friesian cross was the least important variable in MLR and other breeds were second last (25) in LR. The CIV PTA ranked between second (MLR) and ninth (GAM) in importance.

**Table 2:** Variable importance ranking for the models of heifer conception

	LR	GAM	MLR
1	Year: 2003	Month: May	Month: June/July
2	Year: 2008	Year: ≥ 2013	CIV PTA
3	Year: 2004	Year: 2011	Age * Month: June/July
4	Month: June/July	Breed: HO	Breed: NR
5	Age * Month: June/July	Year: 2005	Month: May
6	Breed: NR	Year: 2012	Breed: MO
7	Year: ≥ 2013	Month: June/July	Age * Month: May
8	CIV PTA	Age	Breed: other
9	Year: 2012	CIV PTA	Breed: NO
10	Month: May	Year: 2009	Breed: HO
11	Year: 2005	Year: 2010	Breed: JE
12	Breed: NO	Year: 2006	Breed: JEX
13	Breed: HO		Breed: FRX
14	Year: 2002		Age
15	Age * Month: May		
16	Breed: JE		
17	Breed: MO		
18	Year: 2009		
19	Year: 2010		
20	Age		
21	Year: 2007		
22	Breed: FRX		
23	Breed: JEX		
24	Year: 2011		
25	Breed: other		
26	Year: 2006		

Legend: *LR* logistic regression model, *GAM* generalised additive model, *MLR* mixed logistic regression model, *CIV* calving interval PTA (days), *FRX* Friesian cross, *HO* Holstein, *JE* Jersey, *JEX* Jersey cross, *MO* Montbeliarde, *NO* Normande, *NR* Norwegian Red

* indicates an interaction between two variables

### Evaluation

**Fig. 1:**
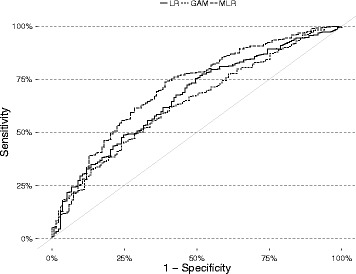
ROC curves for each of the models of heifer conception. Legend: LR = fixed effects logistic regression, GAM = generalised additive model, MLR = mixed effects logistic regression

Results of the discrimination tests are presented in Table 3. Using the standard 50% discrimination threshold, all three models had very high sensitivity, ranging from 97.2% (LR) to 100.0% (GAM). Specificity was very low, ranging from 0.0% (GAM) to 8.3% (MLR). Overall prediction accuracy was between 77.1% (LR) and 78.9% (MLR). The models all had high PPV, indicating that most of the breeding events predicted to result in conception actually did so. NPV ranged from 0.0% (GAM) to 73.3% (MLR), indicating the proportion of services predicted to fail which actually did fail. The Matthews correlation coefficient for the GAM was 0, indicating that its predictions were no better than random. LR and MLR were somewhat better, with MCC of 0.1 and 0.2. Altering the discrimination threshold to minimise false positives and negatives did not greatly alter the sensitivity or specificity of LR or GAM. However, the specificity of MLR improved to 10.5% and the MCC of LR declined to −0.02, while GAM and MLR improved to 0.17 and 0.21. The ROC curves for each model are shown in Fig. 1. All three models performed better than the diagonal line signifying a random predictor. The MLR model was consistently the furthest above the diagonal. At 0.71, the AUC for MLR was the highest of the three models.

**Table 3:** Results of discrimination tests for the models of heifer conception

Model	LR	GAM	MLR
Standard 50% threshold (%)			
Sensitivity	97.20	100.00	99.14
Specificity	6.77	0.00	8.27
PPV	78.43	77.72	79.04
NPV	40.91	0.00	73.33
Accuracy	77.05	77.72	78.89
Matthews correlation coefficient	0.09	0.00	0.20
Optimal thresholds (%)	35.12	60.47	52.63
Sensitivity	99.78	99.57	98.71
Specificity	0.00	5.26	10.53
PPV	77.68	78.57	79.38
NPV	0.00	77.78	70.00
Accuracy	77.55	78.56	79.06
Matthews correlation coefficient	−0.02	0.17	0.21
AUC	0.66	0.64	0.71

Legend: *LR* logistic regression model, *GAM* generalised additive model, *MLR* mixed logistic regression model, *PPV* positive predictive value, *NPV* negative predictive value, *AUC* area under the ROC curve

**Fig. 2:**
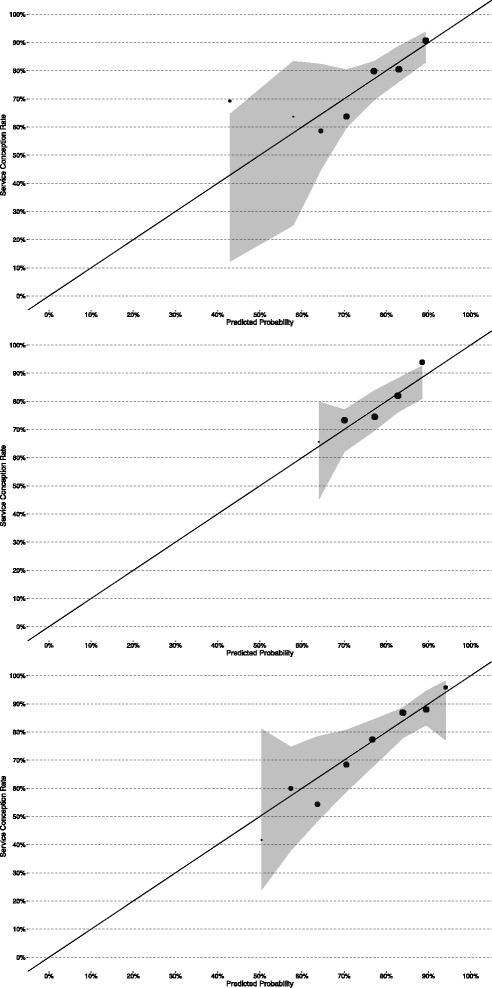
Calibration plots for the models of heifer conception. Legend: Models top – bottom: logistic regression, generalised additive model, mixed logistic regression. The number of services in each group is indicated by the size of the point

Calibration results are presented in Table 4. Fig. 2 display the calibration plots for the three models. All three models were well-calibrated. LR had one small group above the upper confidence interval, indicating some underprediction of the group’s average conception probability, while GAM had one larger group above the confidence interval. All MLR groups were within the confidence interval bounding well-calibrated predictions. The unreliability test found no significant differences between the calibration lines and the diagonal representing perfect prediction of the grouped probabilities. All three models passed the Hosmer-Lemeshow test with *P* > 0.05, indicating no significant difference between the observed and predicted proportions of services resulting in conception. LR had the highest MACE (3.76%) while MLR had the lowest (2.98%). This indicates that, on average, the models were capable of predicting the probability of a service resulting in conception with a high degree of accuracy.

**Table 4:** Results of calibration tests for the models of heifer conception

Model	LR	GAM	MLR
HL *p*-value	0.58	0.73	0.82
MACE (%)	3.76	3.05	2.98
Unreliability p-value	0.80	0.61	0.59

Legend: *LR* logistic regression model, *GAM* generalised additive model, *MLR* mixed logistic regression model, *HL* Hosmer-Lemshow goodness-of-fit test, *MACE* mean absolute calibration error

## Discussion

To the best of the authors’ knowledge, this is the first presentation of a comprehensively-evaluated predictive model of heifer conception to service. It is also the first known description of generalised additive modelling as a modelling technique for this application. In addition to the discrimination methods commonly used to evaluate data mining results, lesser-known calibration techniques were used to measure the accuracy of the predicted probabilities. To minimise the likelihood of bias and overfitting, data splitting and bootstrapping were employed to train and test the models.

The variables included in the models agree with previous findings. Ettema and Santos [2] found that conception rates in groups of maiden heifers calving at older than 750 days were lower than those of younger heifers. Kuhn et al. [13] observed the maximum conception rate at 15 months of age, with month discretised as a categorical variable. Heifers aged 26 months at breeding were 13% less likely to conceive than those aged 15 months. The present study’s predicted conception rate declined linearly with increasing age and interacted with the month of service. Services performed in April had a 0.5% higher probability of conception to service for 19-month-old heifers (the 95th age percentile in our dataset) than for 15-month-olds, while probabilities of conception for services conducted in May and later months were 4.5% and 14.4% lower, respectively. Second- and third-order polynomial functions of age were also considered, but none improved the model and the untransformed age was confirmed to be linear in respect to the probability of conception. In contrast to these findings, age was not significant in the model of conception to first service created by Donovan et al. [9] or the model of conception during a breeding season built by Holm et al. [14].

All of the services in this study were performed within a short breeding season from April to July, removing the utility of analysing the effect of season. However, calendar month was found to be significant, with later months resulting in a higher probability of conception for services with otherwise identical traits. The probability of conception to first service was higher for services performed during the summer in the study of Donovan et al. Kuhn et al. found month to be significant but grouping by season resulted in more distinct variation. The age by calendar month interaction was similar to the interaction between age and season identified by Ettema and Santos [2].

Breed was not identified in any previous multivariate models of conception, which were all carried out using various strains of Holstein heifers. The only genetic traits that have been studied in relation to heifer conception are merit for milk yield and milk composition [12]. Genetic selection for increased milk yield has been linked with a reduction in genetic merit for fertility [31]. In the present study, decreased CIV PTA (i.e. a genetic propensity for shorter calving intervals) resulted in an increased probability of conception. This also agrees with the various indicators of improved reproductive efficiency identified by Cummins et al. [32].

Previous studies modelling heifer fertility did not focus on predictive ability. The AUC values for various models of pregnancy within the heifer’s first breeding season [14] ranged from 0.51 to 0.67, similar to the present study’s range of 0.64 to 0.71. Donovan et al. [9] calculated the Hosmer-Lemeshow goodness-of-fit test. Both of these previous studies [9, 14] carried out the evaluation on the same data used to train the model. Using external data, where possible, or splitting a dataset into training and testing sets are useful strategies to reduce the impact of overfitting [15].

The overall classification accuracy of each of this study’s models was good at over 75%, but all three had low specificity and missed a large proportion of the negative services. For predicting the conception outcome of a single service, high specificity is important to avoid false positives, particularly as the conception rate in dairy heifers is typically high. As the specificity of MLR with the optimal discrimination threshold (52.63%) was significantly higher, this model is recommended as the most useful for decision-support. The on-farm use of the model could consist of ranking the heifers by their probability to conceive during oestrus and selecting the best heifers for more expensive treatments such as high genetic merit sires or sexed semen. Another potential decision-support application is the comparison of likelihoods at potential breeding times for individual heifers.

For events of a stochastic nature, absolute outcomes are not typically desired. Instead, the probability of the event is predicted and used to compare events with different characteristics or to determine an ultimate outcome with random number generation. In this case, accurate prediction of the event probability (i.e. good calibration) is more important than discrimination ability. Stochastic simulation of breeding requires accurate prediction of the probability of conception to correctly model overall reproductive behaviour. The three models performed similarly in most calibration tests. The group of values above the confidence interval in GAM was evidence of some poor accuracy in that probability region. In addition to its better discrimination performance, MLR had well-calibrated predictions and low average error. Its range of predicted probabilities was the widest of the three models. This model was capable of predicting the probability of insemination to service with a high degree of accuracy and is the most suitable model for simulating heifer breeding. Odds ratios for MLR are provided in Table 5.

**Table 5:** Odds ratios for the mixed logistic regression model of heifer conception

Variable	Odds Ratio (95% CI)	*P*-value
Breed		0.53
FR	1	
FRX	1.21 (0.36–4.09)	
HO	1.19 (0.71–1.98)	
JE	0.85 (0.37–1.97)	
JEX	1.15 (0.53–2.50)	
MO	0.64 (0.28–1.47)	
NO	1.79 (0.35–9.10)	
NR	0.47 (0.22–1.02)	
Other	0.64 (0.21–1.96)	
Age (d/10)	1.00 (0.96–1.04)	^1^
CIV	0.93 (0.87–0.99)	0.03
Month		^1^
April	1	
May	7.70 (0.58–101.92)	
June/July	146.98 (2.26–9560.94)	
Age * Month		0.10
April	1	
May	0.97 (0.92–1.02)	
June/July	0.92 (0.84–0.99)	

Legend: *FR* Friesian, *FRX* Friesian cross, *HO* Holstein, *JE* Jersey, *JEX* Jersey cross, *MO* Montbeliarde, *NO* Normande, *NR* Norwegian Red, *CIV* calving interval PTA

Footnote 1: *P*-values are not calculated for the inclusion of individual effects where they are also involved in an interaction

The accuracy of our models may be limited due to the nature of the data available for this study. Key assumptions were made that the heifers involved were truly pubertal before the breeding period, and that service events were performed at a true oestrus event. Hormonal tests to confirm ovarian activity are rarely carried out on commercial dairy farms. The results of the model pertain only to AI services and may not accurately represent the outcomes of natural bull services, which are typically carried out after an unsuccessful period of AI breeding. Further evaluation using bull service data would be needed to assess the predictive ability of our model for natural services. The proposed utility of this model will be to simulate the performance of heifers within a whole-farm decision-support tool.

As described earlier, weight and BCS have been identified as factors that affect heifer fertility. Although neither has been significant in previous model-building [9, 14] and age is closely correlated with breeding weight [11], including them may potentially improve predictive ability. To facilitate this, weight and BCS recorded in a large study and/or measured regularly by commercial dairy farmers would be required. Records of clinical disease could also enhance the model. As shown in a similar study of lactating dairy cows [33], a more comprehensive model capable of predicting a wide range of probabilities has potential as a useful decision-support tool.

The MLR model includes the random effects of herd and year, which should account for potential differences in management. These may differ substantially between herds, but the model can predict the average pasture-based herd. The average herd conception rate could be calculated to estimate its difference from the average. If adequate historical data were available, the model could be recalibrated to fit a single herd.

The factors included in the models are within the capabilities of all farmers to record. The mixed regression model is interpretable and highly accurate at predicting the probability of conception to service in dairy heifers. Given that the model’s performance in discrimination tests was poorer than in calibration tests, it is more suitable for simulation or overall herd prediction than for individual heifer decision-support. Because the findings are consistent with international literature, it is probable that the models are transferable to dairy systems with reproductive management similar to the typical Irish dairy farm [34, 35].

## Conclusions

The risk factors contained in the final mixed logistic regression model to predict the probability of conception to service in seasonal-calving pasture-based dairy heifers include variables related to age, time of service, and genetics. Relevant interactions were also studied. The findings corroborated the results of previous studies of similar systems and combined them in a multivariate model.

Our study also successfully demonstrated the benefits of evaluating the predictive ability of regression models with calibration tests. We suggest that the methods described be used, alongside traditional discrimination tests, to evaluate future models of epidemiological outcomes.

Lastly, the mixed regression model, which is based on easily recorded or simulated data, provides an accurate estimate of the probability of conception. This model will be valuable for the simulation of dairy heifer fertility in seasonal-calving pasture-based dairy production systems.
